# LncRNA MIAT Upregulates NEGR1 by Competing for miR-150-5p as a Competitive Endogenous RNA in SCIRI Rats

**DOI:** 10.1155/2022/2942633

**Published:** 2022-12-28

**Authors:** Zheng Wang, Jianguang Liu, Qiuxiang Yang, Mengjie Ma

**Affiliations:** ^1^Department of Neurology, Wuhan Third Hospital (Tongren Hospital of Wuhan University), Wuhan, China; ^2^Department of Pharmacy, Wuhan Third Hospital (Tongren Hospital of Wuhan University), Wuhan, China; ^3^Department of Gastroenterology and Hepatology, Wuhan Third Hospital (Tongren Hospital of Wuhan University), Wuhan, China

## Abstract

**Objective:**

Spinal cord ischemia–reperfusion injury (SCIRI) can cause a pathological state of irreversible delayed death of neurons in the spinal cord tissue and a range of complications, such as spinal cord dysfunction and motor function impairment. This study aimed to determine whether the long-stranded non-coding ribonucleic acid (lncRNA), myocardial infarction-associated transcript (MIAT), could upregulate neuronal growth regulator 1 (NEGR1) by competing for miR-150-5p as a competitive endogenous RNA in a rat SCIRI model.

**Methods:**

The MIAT knockdown vector or the corresponding blank vector was injected into the spinal cord of healthy sprague Dawley (SD) rats. Administration of the MIAT knockdown vector led to the establishment of the SCIRI rat model. Basso, Beattie & Bresnahan locomotor rating scale (BBB) assessment of hind limb motion. Pathological changes in the spinal cord were observed via hematoxylin and eosin staining and eosin staining. Quantitative polymerase chain reaction was performed to determine the expression levels of the candidate microRNAs and predicted candidate genes, and the relationship between them. Terminal deoxynucleotidyl transferase-mediated dUTP-biotin nick end labeling assay (TUNEL) staining was used to detect apoptosis in the spinal cord tissue of rats in each group. Western blotting was performed to determine the expression of the apoptosis-related proteins, caspase-9, caspase-3, and BCL2-Associated X (Bax)/B-cell lymphoma-2 (Bcl-2). The luciferase reporter gene was used to assess the interaction among the lncRNA, MIAT, and miR-150-5, and the interaction between miR-150-5 and NEGR1.

**Results:**

The sh-lncRNA, MIAT, improved exercise status, and pathological changes in the spinal cord of SCIRI rats, inhibited apoptosis, increased the expression of miR-150-5p, and reduced the expression of NEGR1. Compared with mimics-NC, the transfection of miR-150-5p significantly decreased the relative fluorescence activity ratio of MIAT 3′-untranslated region (3′-UTR) wild-type Human embryonic kidney cells 293 (HEK-293 cells). Compared with mimics-negative control (NC), the transfection of miR-150-5p significantly decreased the relative fluorescence activity ratio of NEGR1 3′-UTR wild-type HEK-293 cells.

**Conclusion:**

MIAT can affect the symptoms of SCIRI in rats. Furthermore, as a competitive endogenous RNA, MIAT upregulates NEGR1 by competing with miR-150-5p in SCIRI rats.

## 1. Introduction

Spinal cord ischemia–reperfusion injury (SCIRI) is a pathological condition in which the degree of ischemic injury is further aggravated by the disappearance of the factors of spinal cord ischemia and hypoxia, re-establishment of blood supply, and even irreversible delayed death of neurons in spinal cord tissue [1], leading to paraplegia, total paralysis, and even death. In recent years, the incidence rate of SCIRI has been increasing, and the treatment of this condition has proven quite difficult. SCIRI can cause a series of complications that can seriously affect the lives of patients [2]. The causes of SCIRI are complex and varied, and include apoptosis, lipid peroxidation, and inflammatory responses. SCIRI is associated with many pathophysiological conditions, such as intraspinal surgery, degenerative cervical myelopathy, and thoracoabdominal or thoracic aneurysm repair surgery [3, 4]. Although previous studies considered cell death during ischemic perfusion as necrosis, recent studies have revealed that apoptosis may be important in its pathogenesis. SCIRI is mainly prevented and treated with drugs and physical therapy; however, a single method cannot prevent or cure this disease, thereby warranting further investigations.

Non-coding ribonucleic acids (ncRNAs) are RNAs that do not encode proteins. In recent years, the expression, function, and mechanism of action of ncRNAs have attracted increasing attention. Based on their molecular size, shape, and function, ncRNAs are classified as ribosomal RNA (rRNA), microRNA (miRNA), circular RNA (circRNA), and long-stranded non-coding RNA (lncRNA) [5]. LncRNAs have been demonstrated to be involved in many biological functions, such as cell proliferation, apoptosis, tumorigenesis, and migration [6–8]. The alteration induced by lncRNAs can cause abnormal expression of disease-associated genes [9]. MiRNAs can interact with lncRNAs to perform their biological functions [10, 11]. In addition, miRNAs and lncRNAs play key roles in the regulation of EZH2 signal transduction. LncRNAs mainly regulate the expression of EZH2 by targeting miRNAs and affect the development of cancer [12]. LncRNAs can also pass through multiple downstream targets, such as STAT3 and NF-*κ*B, PTEN, PI3K/Akt, and miRNAs, which regulate the proliferation and metastasis of prostate cancer cells [13]. Myocardial infarction-associated transcript (MIAT) encodes a spliced lncRNA that may constitute a component of the nuclear matrix. Altered expression of MIAT is associated with the susceptibility to myocardial infarction [14]. Based on recent studies, MIAT is involved in the mechanism of human malignant tumorigenesis [15–17]. However, the expression of MIAT in SCIRI and the underlying mechanisms have not been fully investigated. Neuronal growth regulator 1 (NEGR1) is a glycolphosphatidylinositol (GPI)-anchored membrane protein that plays a role in neuronal cell adhesion, synapse growth, and synapse formation as a neuronal cell adhesion component [18]. To develop more effective and promising therapeutics for SCIRI, how various ncRNAs mediate the pathophysiological mechanisms of SCIRI should be better understood.

In this experiment, healthy sprague Dawley (SD) rats were administered the MIAT knockdown vector or corresponding blank vector via the spinal cord to establish the SCIRI rat model to determine the effect on SCIRI and verify whether MIAT competitively inhibits the binding of miR-150-5p to NEGR1.

## 2. Materials and Methods

### 2.1. Reagents and Antibodies

**Figure 1:**
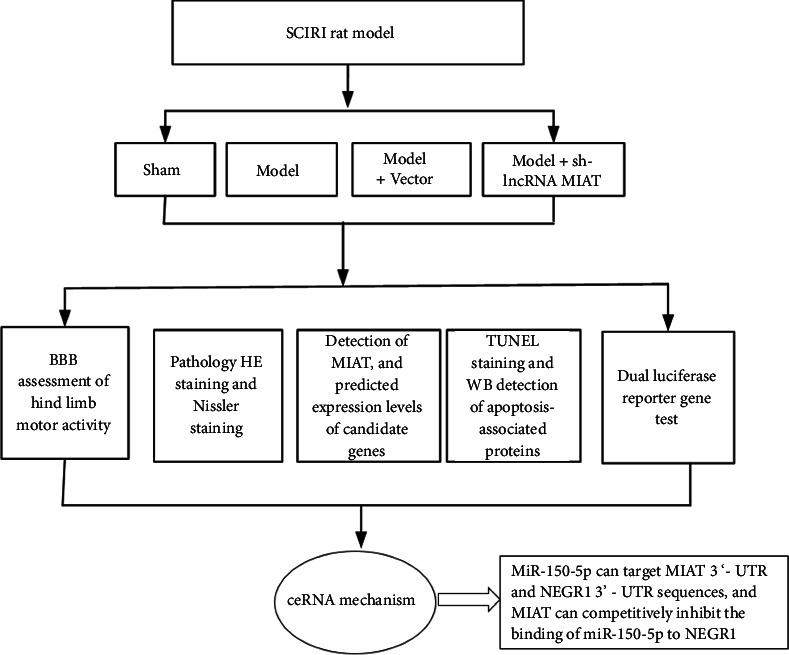
Flowchart of this study.

The Plasmid Extraction Kit (D6950-02, Omega Bio-Tek, Norcross, GA, USA), RNAi-Mate Transfection Reagent (Gima Genetics, China, G04001), phosphate buffer saline (PBS) (Solarbio, China, P1010), sodium pentobarbital (P3761, Sigma Merck, Germany), eosin (E8090, Solarbio, China), hematoxylin (G1004, Servicebio, China), neutral resin (G8590, Solarbio, China), nisin staining solution (G1036, Servicebio, China), TRIzol (15596026, Ambion, USA), SYBR FAST quantitative polymerase chain reaction Master Mix (KM4101, KAPA Biosystems, China), Oligo (dT) 18 Primer (3806, TAKARA, Japan), PrimeScript II Rtase (TAKARA, Japan, 2690A), Recombinant Rnase Inhibitor (TAKARA, Japan, 2313A), 10 mM dNTP Mix (PC2200, Solarbio, China), In Situ Cell Death Detection Kit (11684817910, ROCHE, Switzerland), DAB Concentrated Kit (DA1010-2 × 3 ml, Solarbio, China), Opti-MEM (31985-062, Gibco), Lipofectamine 2000 (11668-027, Invitrogen, USA), Dual luciferase reporter gene assay kit (RG027, Biyuntian, China), caspase-9 (PAB40626, Bioswamp, China), caspase-3 (PAB30047, Bioswamp, China), BCL2-Associated X (Bax) (PAB46088, Bioswamp, China), B-cell lymphoma-2 (Bcl-2)(PAB30041, Bioswamp, China), glyceraldehyde-3phosphate dehydrogenase (GAPDH) (PAB36269, Bioswamp, China), and Goat anti-Rabbit IgG (SAB43714, Bioswamp, China) were employed in this study. A flowchart of this study is shown in Figure 1.

### 2.2. Construction and Infection of the sh-lncRNA MIAT Lentivirus

The human embryonic kidney cell line 293T (Bioswamp Cell Bank, China) served as the packaging cells. Recombinant shuttle and packaging plasmids pGag/Pol, pRev, and pVSV-G were obtained from the Molecular Laboratory of Wuhan Hualianke Biotechnology Co. Ltd., Wuhan, China. The cell suspension was first diluted to 1000 cells/mL with serum-free medium, and 0.1 mL of 0.4% Typanlane solution was added to 0.1 mL of the cell suspension for live cell counting. Cell culture was then performed for 2 days, and a total of 1 × 10^6^/mL of cells were prepared using cell culture medium to freeze the cell lines. The cells were then resuspended and passaged for viral packaging. After virus collection, fluorescent cells were counted using fluorescence microscopy or fluorescence-activated cell sorting, and the virus titer was calculated, accounting for the dilution. Subsequently, infection was carried out. The control group, sh-lncRNA MIAT group, and negative control group were established. First, pre-experiments were conducted to map the multiplicity of infection (MOI) for viral infection. Cells were inoculated in six-well plates 1 day before infection, with 3 × 10^5^ cells/well and 2 mL per well. The virus was thawed on ice, and the original medium was aspirated from the cells. Thereafter, 1 mL of fresh medium was added, followed by 20 *μ*L (MOI = 10) virus. The cells were incubated in an incubator, and the infected medium was replaced with 2 mL of fresh medium after 8 hours of incubation. At 72 hours after cell infection, fluorescent expression was observed and photographed using a fluorescent microscope. Thereafter, samples were collected for subsequent testing.

### 2.3. Construction of the SCIRI Rat Model and Experimental Treatment

Sixty-six 8-week-old male SD rats were selected and housed under specific pathogen-free conditions. All animals were obtained from the Hunan Slaughter Jingda Laboratory Animal Co, Ltd., Changsha, China. The animals were molded after seven days of acclimatization. The animals were divided into four groups. The Sham group comprised nine animals that were randomly selected, weighed, and anesthetized with 3% pentobarbital sodium (30 mg/kg) via intraperitoneal injection. The rats were shaved and skinned, and placed in the supine position. A mid-abdominal incision was performed under aseptic conditions, and the abdominal cavity was closed layer by layer. The Model group comprised nine animals that were weighed and anesthetized via an intraperitoneal injection of 3% pentobarbital sodium (30 mg/kg). The rats were shaved and placed in the supine position. A median abdominal incision was then established under aseptic conditions. After the surrounding soft tissues were separated, the abdominal aorta below the left kidney was clamped using the artery, with the blood supply restored after 45 minutes. Finally, the abdominal cavity was closed layer by layer. The Model + Vector group comprised nine rats that were administered the MIAT blank vector (0.5 mg/kg) via the spinal cord at 45 minutes before clamping of the abdominal aorta. The Model + sh-lncRNA MIAT group comprised nine rats that were administered the MIAT knockdown virus (6 *μ*L) via the spinal cord at 45 minutes before clamping of the abdominal aorta. After 48 hours, the rats were anesthetized with 3% pentobarbital sodium, and the spinal cord was retrieved from the lumbar vertebrae. All laboratory operators possess the “Certificate of Competence in Laboratory Animal Technology Examination.” All animal experiments conformed with the relevant regulations of the Hubei Provincial Animal Management Committee “Ethics Certificate for Laboratory Animals” (Experimental animal use license number: SYXK (E) 2018-0104; certificate of conformity no. 430727211102234258; and facility certificate no. 430727213360787283).

### 2.4. Basso, Beattie, and Bresnahan Score

The motor activity of the hind limbs of rats was assessed 12, 24, and 48 hours after surgery according to the Basso, Beattie, and Bresnahan (BBB) motor rating scale, which ranges from 0 (complete paralysis) to 21 (normal movement). The reference standards used are listed in Table 1.

### 2.5. Hematoxylin and Eosin Staining

Fixed rat spinal cord tissues were dewatered and embedded in wax according to the conventional procedure. The tissue sections (3 *μ*m) were sliced, spread, baked, and then stained with hematoxylin and eosin (HE). Tissue sections were dewaxed in water. After washing with water and staining with hematoxylin for 3–6 minutes, 1% hydrochloric acid alcohol 1–3 seconds. After washing with water, the blue-promoting solution appeared blue after 5–10 seconds. 0.5% eosin solution was added for 2–3 minutes after washing with water. The following solutions were added to the section after washing with water: 80% ethanol, 15–30 seconds; 95% ethanol, 15–30 seconds; anhydrous ethanol, 1–2 seconds; xylene (I), 2–3 seconds; and xylene (II), 2–3 seconds. After rinsing with water, neutral tree sealant was added to the sample. Finally, the sample was photographed using a microscope, and the relevant areas of the sample were captured using the Leica Application Suite graphics system.

### 2.6. Nylon Dyeing

A 0.3-cm-thick tissue block containing the hippocampus and a brain tissue block containing the frontal lobe were cut from the fixed rat spinal cord tissue, de-watered, waxed, and embedded according to the usual procedure. After the tissue block was sectioned (3 *μ*m), the slices were spread, baked, and stained with nylon. The tissue sections were routinely dewaxed in water, and placed in nisin staining solution for 2–5 minutes. The brain tissue was differentiated until nichrome appeared dark blue with a light blue or colorless background. The sections were oven dried at 65°C and sealed with neutral gum. The sample was photographed using a microscope, and the relevant areas of the sample were captured using the Leica Application Suite graphics system.

### 2.7. qRT-PCR

The cells (1 × 10^6^) were precipitated in a 1 mL TRIzol homogenizer tube, homogenized in a homogenizer for 20 seconds, and subjected to total RNA extraction. Reverse transcription was then performed, and the prepared cDNA was amplified via PCR. The following cycling program was employed: pre-denaturation 95°C for 3 minutes; 95°C for 5 seconds, 56°C for 10 seconds, and 72°C for 25 seconds (40 cycles). The relative mRNA content was calculated using the 2^−*ΔΔ*t^ method. The primers used were as follows: MIAT-F, AGAAGGAGGAGGCAGATAGTT; MIAT-R, TGCTGAGTTGTGGTGACG; miR-150-5p-F, GGGTCTCCCAACCCTTGTA; miR-150-5p-R, CTGGTGTCGTGGAGTCGG; NEGR1-F, GTGTTACTTGGAAGACGGAGC; NEGR1-R, GCCATCATCTGTCACATCAAC.

GAPDH-F, CAAGTTCAACGGCACAG; GAPDH-R, CCAGTAGACTCCACGACAT; U6-F, CTCGCTTCGGCAGCACATATACT; U6-R, ACGCTTCACGAATTTGCGTGTC. The PCR primers were synthesized by Wuhan Tianyi Huayu Gene Technology Co, Ltd., Wuhan, China.

### 2.8. TUNEL Staining

Fixed rat spinal cord tissues were dewatered and embedded in wax according to the conventional procedure. Slices (3 *μ*m) were cut, spread, baked, and stained with terminal deoxynucleotidyl transferase-mediated dUTP-biotin nick end labeling assay (TUNEL). The slides were then baked in a constant temperature oven at 65°C for 1 hour and soaked in xylene I for 15 minutes followed by xylene II for 15 minutes. The dewaxed sections were soaked in 100% alcohol, 95% alcohol, 85% alcohol, and 75% alcohol for 5 minutes and rinsed with tap water for 10 minutes. After the sections were incubated with proteinase K working solution at 37°C for 15 minutes, 50 *μ*L of the TUNEL reaction mix was added to the section, which was incubated in a covered wet box placed in a dark room at 37°C for 60 minutes. After the samples were rinsed, 50 *μ*L of transformed POD was added to the sample, which was incubated in a wet box at 37°C for 30 minutes. The samples were rinsed, treated with 50 *μ*L of DAB substrate, incubated at room temperature for 10 minutes, rinsed, re-stained with hematoxylin, dehydrated, and transparent. The nuclei of apoptotic cells were stained brownish-yellow or gray, whereas the nuclei of non-apoptotic cells were stained blue.

### 2.9. Western Blot

Spinal cord tissue was added to the lysate (containing protease and phosphatase inhibitors) at 200 *μ*L lysate per 20 mg tissue for protein extraction. The protein concentration of each group was measured using a BCA Kit (solarbio, China), and 20 *μ*g of protein was added to the gel (formulated with 12% isolate and 5% concentrate). Select concentrated gel 80 V for 40 minutes, separated gel 120 V for 50 minutes, constant pressure 90 V for 50 minutes, and 5% skimmed milk powder closed at room temperature overnight at 4°C. The membrane was incubated with primary antibodies against caspase-9 (1 : 1000), caspase-3 (1 : 1000), Bax (1 : 1000), Bcl-2 (1 : 1000), and GAPDH (1 : 1000) at room temperature for 1 hour. After the membrane was washed, it was incubated with the secondary antibody, goat anti-rabbit IgG (1 : 20,000), at room temperature for 1 hour. The enhanced chemiluminescence (ECL) luminescent solution was added to the membrane, which placed in a fully automated chemiluminescent analyzer for color development. The grayscale values of the relevant bands were read using the TANON GIS software. Three replicates were used for each group.

### 2.10. Cell Lines

Human embryonic kidney HEK-293 cells were obtained from the Shanghai Cell Bank of the Chinese Academy of Sciences, Shanghai, China. Frozen cells were removed from the liquid nitrogen tank and transferred to a 37°C water bath. After the cells were thawed, the cell suspension was aspirated into a centrifuge tube. Following the addition of 4 mL of complete medium, the sample was centrifuged at 400*g* for 3 minutes, and the resulting supernatant was discarded. The cells were resuspended in 1 mL of medium and transferred to culture flasks. After 4 mL of complete medium was added, the flasks were incubated at 37°C with 5% CO_2_.

### 2.11. Construction and Transfection of the Luciferase Plasmids

Briefly, wt-NEGR1 3′-untranslated region (3′-UTR) and MT-NEGR1 3′-UTR were synthesized. The pmirGLO vector was used for the cloning. The vector was digested with the target gene at 37°C for 1–2 hours. Electrophoretic detection of the digested product and its recovery was performed according to the kit instructions. The vector was ligated to the target gene using T4 DNA ligase at 16°C overnight. Thereafter, the transformation experiment was carried out at 37°C. Of note, the spots could appear after 12–16 hours. Several monoclonal shake bacteria were randomly selected and sent to a sequencing company for sequencing verification. Thereafter, positive clones were selected. Transfection was performed according to the following grouping: EGR1 3′-UTR wild-type (miR-150-5p-NC, miR-150-5p-mimics, miR-150-5p-NC + interfering MIAT vector, miR-150-5p-mimics + interfering MIAT vector, and miR-150-5p-mimics + negative control), NEGR1 3′-UTR mutant (miR-150-5p-NC, miR-150-5p-mimics, miR-150-5p-NC + interfering MIAT vector, miR-150-5p + interfering MIAT vector, and miR-150-5p-mimics + negative control). At 24 hours before the transfection, 5 × 10^3^ cells were inoculated in 100 *μ*L complete medium and seeded in 96-well plates with 90% cell fusion at the time of transfection. Thereafter, 0.2 *μ*g plasmid DNA and 5 pmol miRNA were diluted with 25 *μ*L Opti-MEM, and 0.25 *μ*L Lipofectamine 2000 was diluted with 25 *μ*L Opti-MEM, mixed, and allowed to stand for 20 minutes at room temperature. Fifty microliters of the complex was added to the wells of the plate containing the cells and 50 *μ*L of fresh medium. The plate was then incubated at 37°C in a 5% CO_2_ incubator. Transfection was allowed to proceed for 4 hours before medium change. After 24 hours of incubation, the subsequent assays were performed.

### 2.12. Dual Luciferase Assay

Cell lysis solution (100 *μ*L) was added to each well, and the cells were fully lysed and transferred to eppendorf (EP) tubes. Sea kidney luciferase assay buffer was added to sea kidney luciferase assay substrate (100×) at a 1 : 100 ratio to prepare the sea kidney luciferase assay working solution, which was detected using a full-function microplate detector, with a detection interval of 1 second, and a detection time of 10 seconds. To detect the value of firefly luciferase activity F (firefly luciferase), 100 *μ*L of firefly luciferase assay reagent was added to each cell sample. The EP tubes were immediately placed in a full-function microplate assay after mixing to calculate the *F* value. Thereafter, 100 *μ*L of sea kidney luciferase assay working solution was added, mixed well, and immediately used in the full-function microplate assay to calculate the sea kidney luciferase activity value *R*. After completion of all sample assays, the relative luciferase activity was calculated based on the detected *F* and *R* values.

### 2.13. Statistical Analysis of Data

All data were statistically analyzed using the SPSS software (version 19.0). The values are expressed as mean ± standard deviation. One-way Analysis of Variance was used for comparison between multiple groups, and least significant difference (LSD) test was used for two-way comparison between means. Differences were considered statistically significant at *P* < 0.05.

## 3. Results

### 3.1. Identification of sh-lncRNA MIAT Lentivirus Infection and Efficiency

**Figure 2:**
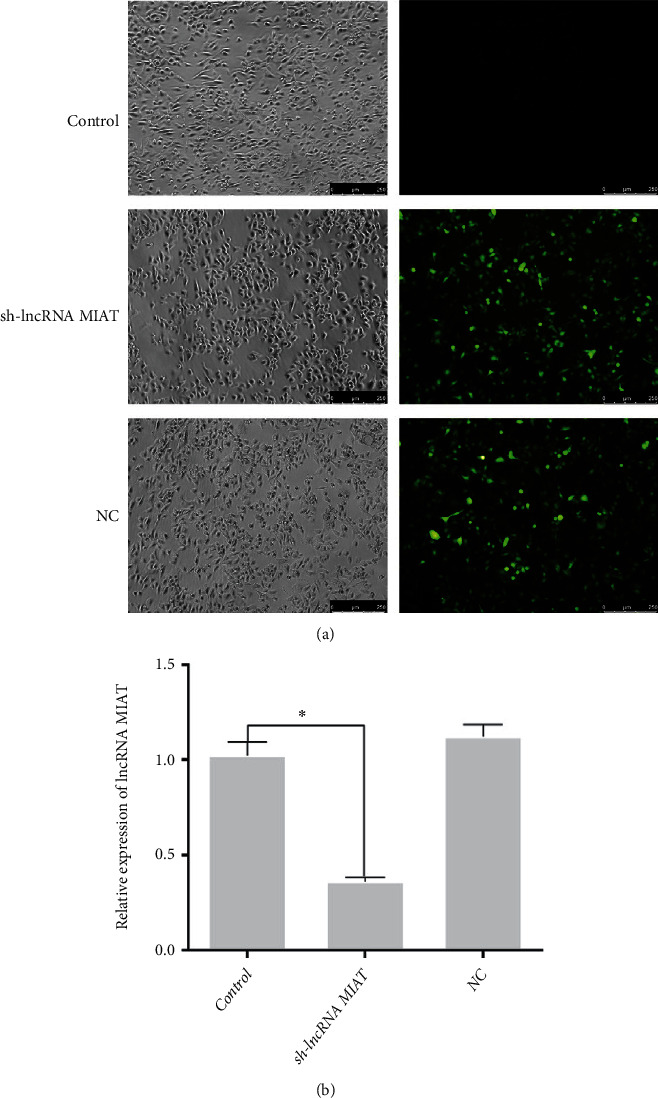
sh-lncRNA MIAT lentivirus infection and efficiency identification. (a) sh-lncRNA MIAT infection. (b) Efficiency identification.

PC-12 cells were infected with the sh-lncRNA MIAT virus for 72 hours, and fluorescent expression was observed under a fluorescent microscope. The results are shown in Figure 2(a). Distinct green fluorescence was observed after infection with sh-lncRNA MIAT virus, with the infection efficiency exceeding 90%. The groups were then identified for infection efficiency (Figure 2(b)), and the relative expression of MIAT was significantly lower in the sh-lncRNA MIAT group than that in the control group (*P* < 0.05). Such finding indicates successful infection with the sh-lncRNA MIAT lentivirus.

### 3.2. Effects of sh-lncRNA MIAT on the Behavior and Spinal Cord Pathology of SCIRI Rats

**Figure 3:**
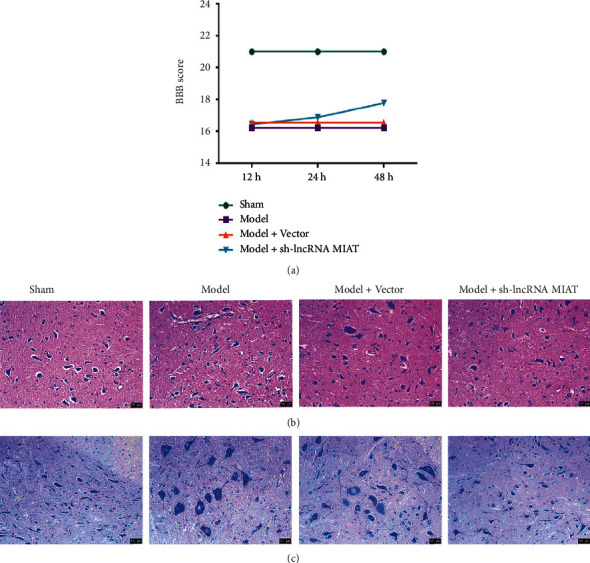
Effects of sh-lncRNA MIAT on the behavior and spinal cord pathology of SCIRI rats. (a) Rat behavior BBB score. (b) HE staining (200×), and (c) Nisin staining (200×).

The motor activity of the hind limbs of each group of rats was assessed at 12, 24, and 48 hours postoperatively, according to the BBB motor quantification scale. The results are shown in Figure 3(a). Sham group rats had no significant change in BBB score at 12, 24, and 48 hours, and ball movement appeared normal. The Model and Model + Vector groups had significantly lower BBB scores and no significant changes in scores at 12, 24, and 48 hours relative to the Sham group. The Model + sh-lncRNA MIAT group had a significant increase in BBB score after 24 hours, with improved hind limb movement. The pathological condition of the spinal cord was observed via HE staining, and the results are shown in Figure 3(b). The spinal cord tissues of rats in the Sham group were structurally intact, with a high number of normal neurons and nuclei with obvious nucleoli. The spinal cord tissue structure was severely damaged in the Model and Model + Vector groups, with a low number of normal neurons and numerous lysed nuclei. In the Model + sh-lncRNA MIAT group, the structure of the spinal cord tissue appeared intact, the number of normal neurons was increased compared with that in the Model group, the nucleus morphology was approximately normal, and the pathological changes were significantly reduced. Neuronal cell morphology was observed under a nylon-stained light microscope (Figure 3(c)). In the Sham group, the gray–white matter of the spinal cord tissue was well arranged, the neuronal structure was normal, and uniform plaque-like staining of the Nisus vesicles was clearly visible. In the Model and Model + Vector groups, the spinal cord tissue was disrupted in the gray matter structure, the white matter appeared lax and edematous, the neurons were degenerated and necrotic, the Nisus vesicles were lysed and disappeared, and cell swelling was more severe. Spinal cord tissue edema was reduced in the Model + sh-lncRNA MIAT group. Furthermore, the swelling of neurons was not obvious, multiple Nisus vesicles were visible in the cytoplasm, and the pathological condition of the spinal cord was significantly reduced. Accordingly, sh-lncRNA MIAT can improve hind limb locomotion and spinal cord pathology in SCIRI rats.

### 3.3. Effect of sh-lncRNA MIAT on Apoptosis in SCIRI Rats

**Figure 4:**
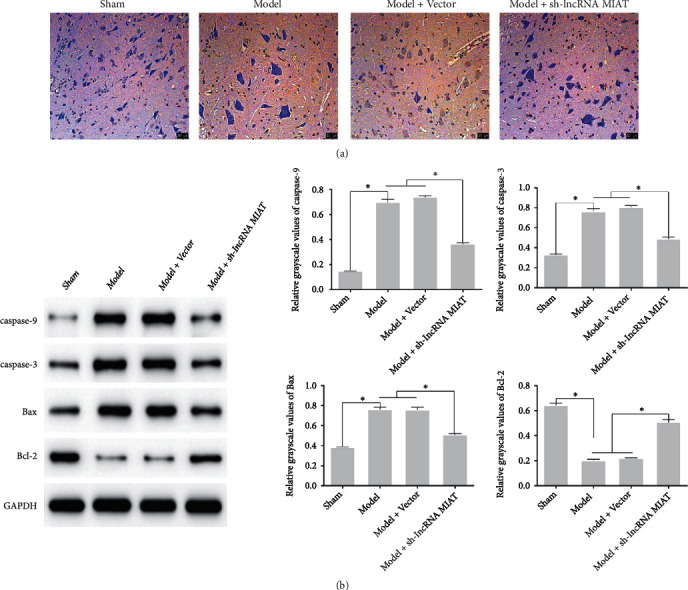
Effect of sh-lncRNA MIAT on apoptosis in SCIRI rats. (a) Apoptosis detection via TUNEL staining. (b) Detection of changes in apoptosis-associated proteins based on western blot.

Cell apoptosis was observed via TUNEL staining; the results are shown in Figure 4(a). The positive apoptotic area was smaller, and the apoptosis rate was lower in the Sham group. Furthermore, numerous brownish-yellow cell nuclei and increased apoptosis were observed in the Model and Model + Vector groups. However, the brownish-yellow area and apoptosis were significantly reduced in the Model + sh-lncRNA MIAT group. The expression of apoptosis-related proteins in each group was detected using western blotting; the results are shown in Figure 4(b). The expression levels of caspase-9, caspase-3, and Bax were significantly higher (*P* < 0.05), and the expression of BcI-2 was significantly lower (*P* < 0.05) in the Model group than that in the Sham group. The expression levels of caspase-9, caspase-3, and Bax were significantly lower (*P* < 0.05), and the expression of BcI-2 was significantly higher (*P* < 0.05) in the Model + sh-lncRNA MIAT group relative to the Model and Model + Vector groups. Therefore, sh-lncRNA MIAT can inhibit apoptosis in SCIRI rats.

### 3.4. MIAT Competitively Inhibits the Binding of miR-150-5p to NEGR1

**Figure 5:**
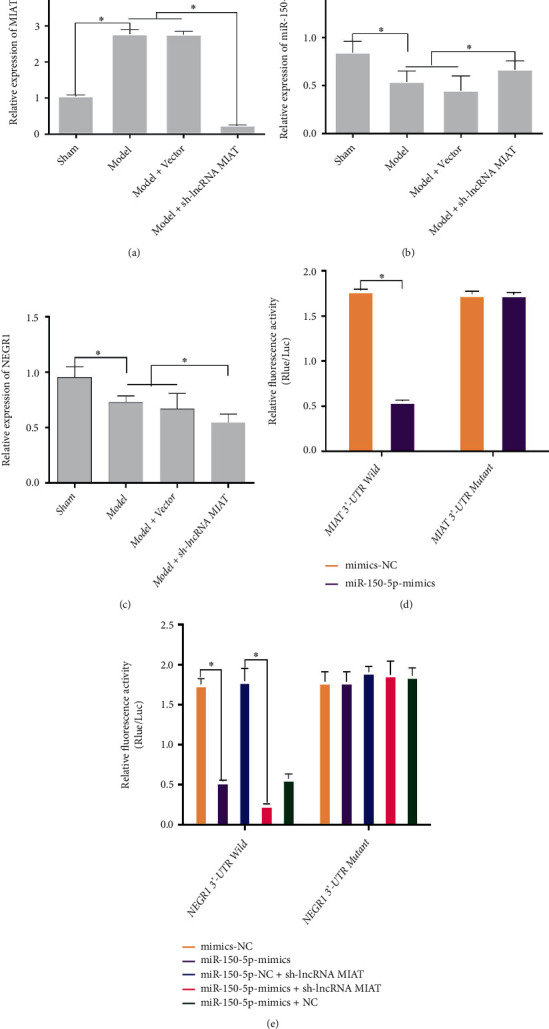
MIAT competitively inhibits the binding of miR-150-5p to NEGR1. (a) Relative expression of MIAT based on qRT-PCR. (b) Relative expression of miR-150-5p based on qRT-PCR. (c) Relative expression of NEGR1 based on qRT-PCR. (d) Interaction between MIAT and miR-150-5p based on the luciferase reporter gene. (e) Interaction between miR-150-5p and NEGR1 based on the luciferase reporter gene.

**Figure 6:**
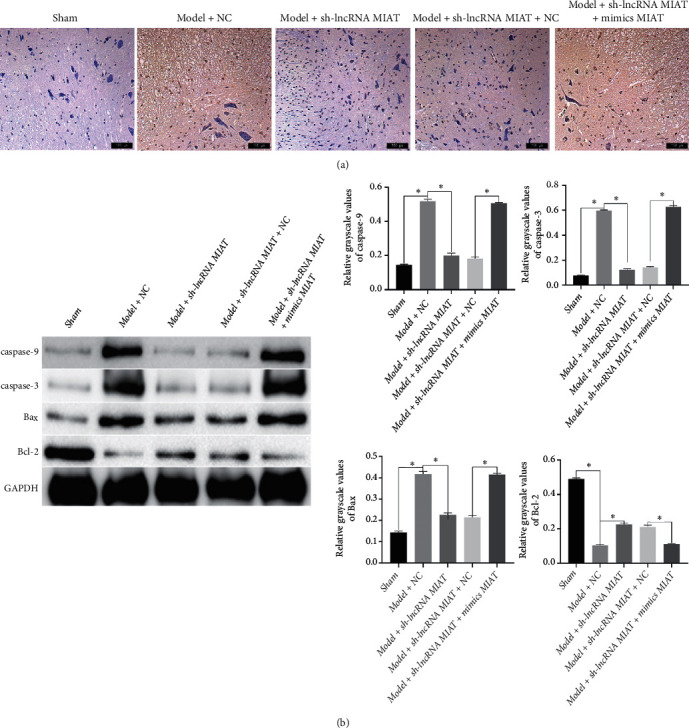
Knockdown of the lncRNA and MIAT was verified via rescue experiments. (a) Apoptosis detection via TUNEL staining. (b) Detection of changes in apoptosis-associated proteins based on western blot.

The expression levels of miR-150-5p and NEGR1 in the spinal cord were determined using quantitative reverse transcriptase PCR (qRT-PCR) (Figures 5(a), 5(b), and 5(c)). The expression level of MIAT was significantly higher (*P* < 0.05), while those of miR-150-5p and NEGR1 were significantly lower (*P* < 0.05) in the Model group than in the Sham group. The expression levels of MIAT and NEGR1 were significantly lower (*P* < 0.05), while that of miR-150-5p was significantly higher (*P* < 0.05) in the Model + sh-lncRNA MIAT group than in the Model and Model + Vector groups. To verify whether MIAT competitively inhibits the binding of miR-150-5p to NEGR1, we examined the interactions between MIAT and miR-150-5p, miR-150-5p, and NEGR1 in cells using a luciferase reporter gene. Transfection with miR-150-5p significantly reduced the relative fluorescence activity ratio of MIAT 3′-UTR wild-type HEK-293 cells compared with mimics-NC (*P* < 0.05; Figure 5(d)). As shown in Figure 5(e), transfection with miR-150-5p significantly reduced the relative fluorescence activity ratio of NEGR1 3′-UTR wild-type HEK-293 cells compared with mimics-NC (*P* < 0.05). Similarly, transfection with miR-150-5p decreased the relative fluorescence activity ratio of NEGR1 3′-UTR wild-type HEK-293 cells compared with mimics-NC + sh-lncRNA MIAT (*P* < 0.05). Accordingly, miR-150-5p can target the binding to the MIAT 3′-UTR and NEGR1 3′-UTR sequences, and MIAT can competitively inhibit the binding of miR-150-5p to NEGR1. The knockdown of the lncRNA MIAT was validated using rescue experiments. As shown in Figure 6(a), the Sham group had fewer apoptotic areas and lower apoptosis. Furthermore, numerous brownish-yellow nuclei and increased apoptosis were observed in the Model + NC group. However, the brownish-yellow area and apoptosis were significantly reduced in the Model + sh-lncRNA MIAT group. Compared with the Model + sh-lncRNA MIATl+NC group, the Model + sh-lncRNA MIAT + mimics MIAT group had numerous brownish-yellow nuclei and increased cell apoptosis. Based on western blot analysis (Figure 6(b)), the expression levels of caspase-9, caspase-3, and Bax were significantly increased while that of BcI-2 was significantly decreased in the Model + NC group compared with the Sham group. Furthermore, the expression levels of caspase-9, caspase-3, and Bax were significantly decreased while that of BcI-2 was significantly increased in the Model + sh-lncRNA MIAT group compared with the Model + NC group. Finally, the expression levels of caspase-9, caspase-3, and Bax was significantly increased while that of BcI-2 was significantly decreased in the Model + sh-lncRNA MIAT + mimics MIAT group compared with the Model + sh-lncRNA MIAT1+NC group.

## 4. Discussion

SCIRI is associated with the risk of neurological dysfunction and paralysis, which seriously affect the quality of life of patients. Currently, no effective treatment is available for SCIRI. Based on increasing studies, many ncRNAs play key roles in the pathophysiology of SCIRI; this is because individual miRNAs can regulate several target genes, affect the entire gene network, and be effectively targeted by different tools to increase the levels of miRNAs with beneficial functions or decrease the levels of pathogenic miRNAs [1, 19]. LncRNAs have been identified as potential oncogenes or oncogenic suppressors [20]. By analyzing the expression pattern of lncRNAs in the SCIRI rat model using high-throughput RNA sequencing, Zhou et al. found that 6,707 mRNAs and 1,455 lncRNAs were differentially expressed, of which 761 lncRNAs were upregulated and 694 lncRNAs were downregulated [21]. Similarly, another study found that 7,980 lncRNAs and 3,428 mRNAs were differentially expressed [22]. These bioinformatics results suggest that these differentially expressed ncRNAs and mRNAs can participate in many bioinformatics pathways and processes, providing new insights into the identification of potential therapeutic targets for SCIRI. Among them, MIAT is widely overexpressed in different tumors and can regulate complex regulatory mechanisms, such as cell proliferation, migration, invasion, and apoptosis [23]. In this study, we replicated the SCIRI rat model by injecting MIAT knockdown vectors with corresponding blank vectors into the rat spinal cord to determine the effects of MIAT on the motor and spinal cord pathological changes in SCIRI rats. Locomotion was identified to be significantly improved in SCIRI rats, and spinal cord pathological changes were significantly alleviated after MIAT knockdown.

MIAT regulates diabetes-induced microvascular dysfunction by competing with endogenous vascular endothelial growth factor and miR-150 in retinal endothelial cells. According to Zhu et al., MIAT promotes the proliferation of melanoma cells by targeting miR-150 [24]. In this study, the expression of miR-150-5p was increased, whereas that of NEGR1 was decreased after MIAT knockdown. EGR1 is an inducible protein that belongs to the early growth response gene family and is associated with cell proliferation, differentiation, and death pathways in vascular cells [25]. Apoptosis can lead to tissue loss in ischemic lesions. Caspase-9 is a key molecule in the mitochondrial pathway of apoptosis. Caspase-9 cleaves effector caspases, such as caspase-3, zymogen, adenosine polydiphosphate polymerase, and other intracellular substrates, ultimately causing apoptosis [26]. In the present study, after MIAT knockdown, the expression levels of caspase-9, caspase-3, and Bax in cells were significantly reduced, whereas that of BcI-2 was significantly increased.

MIAT regulates genes at the transcriptional and post-transcriptional levels. At the transcriptional level, MIAT exerts regulatory effects in the nucleus through interaction with nuclear factors, whereas at the post-transcriptional level, it exerts regulatory effects in the cytoplasm through competitive endogenous RNA (ceRNA). Zhang et al. [15] demonstrated that MIAT promotes cell invasion via miR-150 in non-small cell lung cancer. Furthermore, the regulation between MIAT/miR-29a-3p/HDAC4 was recognized to be involved in cell biological behavior and the development of gastric cancer [16]. In this study, a luciferase reporter gene was used to confirm whether MIAT competitively inhibited the binding of miR-150-5p to NEGR1. MiR-150-5p could target binding to the MIAT 3′-UTR and NEGR1 3′-UTR sequences, and MIAT could competitively inhibit the binding of miR-150-5p to NEGR1. Many studies have demonstrated that MIAT is a pro-oncogene that acts as a ceRNA network component to regulate VEGF/Akt levels by forming miR-150-5p in retinal endothelial cells [27, 28]. MIAT is also a potential biomarker of chronic lymphocytic leukemia aggressiveness [29].

## 5. Conclusion

**Figure 7:**
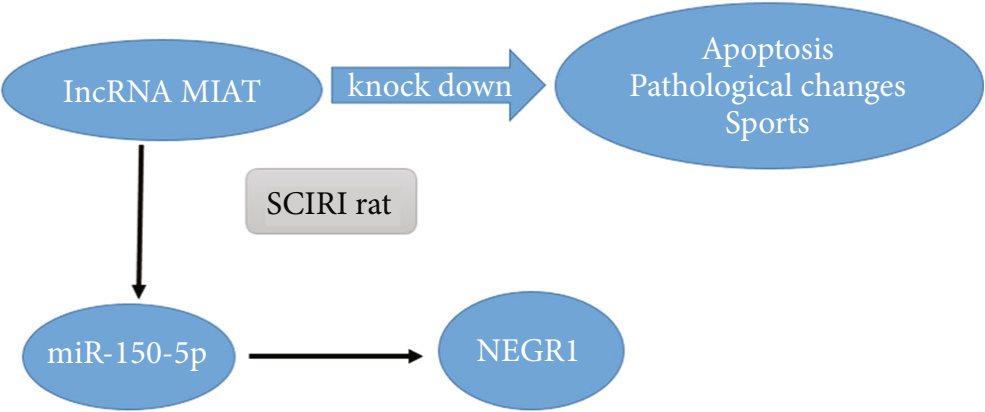
Summary of the results of the study.

In this study, MIAT knockdown was found to inhibit the apoptosis of SCIRI cells, improve the motor status of rats, and improve pathological changes in the spinal cord. Furthermore, the lncRNA, MIAT, upregulated NEGR1 as a competing endogenous RNA in SCIRI rats by competing with miR-150-5p. A summary of the results is provided in Figure 7. Collectively, our findings help us fully understand the mechanistic involvement of lncRNAs in SCIRI.

## Figures and Tables

**Table 1:** BBB Sport Rating Scale.

Score	Description
0–7: Assessment of hind limb joint motion in early response	
1	Slight movement of one or both joints, usually hip/knee
2	Extensive movement of one joint, or light movement of other joints
3	Extensive movement of both joints
4	Slight movement of three joints of the hind limbs
5	Slight movement of two joints, and slight movement of another joint
6	Extensive movement of two joints, and slight movement of the other joint
7	Extensive movement of the three joints of the hind limbs
8–13: Assessment of gait and coordinated movement in mid-response	
8	Weightless dragging or foot placed in weightless position
9	Plantar in weight-bearing position only, or occasional/frequent/continuous dorsal foot-bearing walking, no plantar walking
10	Occasional weight-bearing walking without coordinated fore and hind limb movements
11	Frequent to continuous weight-bearing walking without coordinated fore and hind limb movements
12	Frequent to sustained weight-bearing walking with occasional coordinated fore and hind limb movements
13	Continuous weight-bearing walking with frequent coordinated anterior and posterior limb movements
14–21: Assessing the fine movements of the paws during exercise	
14	Continuous coordinated gait; continuous front and hind limb motor coordination; dominant paw rotation; or frequent plantar walk during movement
15	Continuous coordinated gait; no or occasional toe extension when forelimbs advance; dominant paws parallel to body when first touching the ground
16	Continuous coordinated gait; frequent toe extensions; dominant paw touches the ground parallel to the body and rotates when lifted
17	Continuous coordinated gait; frequent toe extensions; dominant paws parallel to the body both when touching the ground and when lifted
18	Continuous coordinated gait; continuous toe extension; dominant paws parallel to the body when touching the ground and rotating when lifted
19	Continuous coordinated gait; continuous toe extension; dominant paws parallel to the body both when touching the ground and when lifted
20	Continuous coordinated gait; continuous toe extension; dominant paws parallel to the body both when touching the ground and when lifted; unstable body and continuous tail up
21	Continuous coordinated gait; continuous toe extension; dominant paws parallel to the body when touching the ground and when lifted; stable body and continuous tail up

## Data Availability

The data used to support the findings of this study are included within the article.

## References

[1] Li R., Zhao K., Ruan Q., Meng C., Yin F. (2020). Bone marrow mesenchymal stem cell-derived exosomal microRNA-124-3p attenuates neurological damage in spinal cord ischemia–reperfusion injury by downregulating Ern1 and promoting M2 macrophage polarization. *Arthritis Research & Therapy*.

[2] Qian X., Wu Y. H., Che Y. Y. (2021). IP(3)R-mediated activation of BK channels contributes to mGluR5-induced protection against spinal cord ischemia-reperfusion injury. *Neurochemistry International*.

[3] Chen F., Li X., Li Z., Zhou Y., Qiang Z., Ma H. (2020). The roles of chemokine (C-X-C motif) ligand 13 in spinal cord ischemia-reperfusion injury in rats. *Brain Research*.

[4] Laliberte A. M., Karadimas S. K., Vidal P. M., Satkunendrarajah K., Fehlings M. G. (2021). Mir21 modulates inflammation and sensorimotor deficits in cervical myelopathy: data from humans and animal models. *Brain Communications*.

[5] Dong Y., Xu W., Liu C., Liu P., Li P., Wang K. (2019). Reactive oxygen species related noncoding RNAs as regulators of cardiovascular diseases. *International Journal of Biological Sciences*.

[6] Wang W., Min L., Qiu X. (2021). Biological function of long non-coding RNA (lncRNA) Xist. *Frontiers in Cell and Developmental Biology*.

[7] Lakshmi S., Hughes T. A., Priya S. (2021). Exosomes and exosomal RNAs in breast cancer: a status update. *European Journal of Cancer*.

[8] Chen J., Zhang K., Zhi Y. (2021). Tumor-derived exosomal miR-19b-3p facilitates M2 macrophage polarization and exosomal LINC00273 secretion to promote lung adenocarcinoma metastasis via hippo pathway. *Clinical and Translational Medicine*.

[9] Wang D., Wang L., Han J., Zhang Z., Fang B., Chen F. (2021). Bioinformatics-based analysis of the lncRNA-miRNA-mRNA network and TF regulatory network to explore the regulation mechanism in spinal cord ischemia/reperfusion injury. *Frontiers in Genetics*.

[10] Ghafouri-Fard S., Glassy M. C., Abak A., Hussen B. M., Niazi V., Taheri M. (2021). The interaction between miRNAs/lncRNAs and notch pathway in human disorders. *Biomedicine & Pharmacotherapy*.

[11] Raei N., Safaralizadeh R., Hesseinpourfeizi M., Yazdanbod A., Pourfarzi F., Latifi-Navid S. (2021). Crosstalk between lncRNAs and miRNAs in gastrointestinal cancer drug resistance. *Life Sciences*.

[12] Mirzaei S., Gholami M. H., Hushmandi K. (2022). The long and short non-coding RNAs modulating EZH2 signaling in cancer. *Journal of Hematology & Oncology*.

[13] Mirzaei S., Paskeh M. D. A., Okina E. (2022). Molecular landscape of lncRNAs in prostate cancer: a focus on pathways and therapeutic targets for intervention. *Journal of Experimental & Clinical Cancer Research*.

[14] Ghafouri-Fard S., Azimi T., Taheri M. (2021). Myocardial infarction associated transcript (MIAT): review of its impact in the tumorigenesis. *Biomedicine & Pharmacotherapy*.

[15] Zhang H. Y., Zheng F. S., Yang W., Lu J. B. (2017). The long non-coding RNA MIAT regulates zinc finger E-box binding homeobox 1 expression by sponging miR-150 and promoting cell invasion in non-small-cell lung cancer. *Gene*.

[16] Yanfeng L., Kuan W., Yuzhe W. (2017). lncRNA-MIAT regulates cell biological behaviors in gastric cancer through a mechanism involving the miR-29a-3p/HDAC4 axis. *Oncology Reports*.

[17] Crea F., Venalainen E., Ci X. (2016). The role of epigenetics and long noncoding RNA MIAT in neuroendocrine prostate cancer. *Epigenomics*.

[18] Cheon Y., Yoo A., Seo H. (2021). Na/K-ATPase beta1-subunit associates with neuronal growth regulator 1 (NEGR1) to participate in intercellular interactions. *BMB Reports*.

[19] Liu Z. G., Li Y., Jiao J. H., Long H., Xin Z. Y., Yang X. Y. (2020). MicroRNA regulatory pattern in spinal cord ischemia–reperfusion injury. *Neural Regeneration Research*.

[20] Kopp F., Mendell J. T. (2018). Functional classification and experimental dissection of long noncoding RNAs. *Cell*.

[21] Zhou Z., Han B., Jin H., Chen A., Zhu L. (2020). Changes in long non-coding RNA transcriptomic profiles after ischemia–reperfusion injury in rat spinal cord. *PeerJ*.

[22] Wang Q., Li Z. X., Li Y. J. (2019). Identification of lncRNA and mRNA expression profiles in rat spinal cords at various time-points following cardiac ischemia/reperfusion. *International Journal of Molecular Medicine*.

[23] Da C. M., Gong C. Y., Nan W., Zhou K. S., Wu Z. L., Zhang H. H. (2020). The role of long non-coding RNA MIAT in cancers. *Biomedicine & Pharmacotherapy*.

[24] Zhu L., Wang Y., Yang C. (2020). Long non-coding RNA MIAT promotes the growth of melanoma via targeting miR-150. *Human Cell*.

[25] Wang B., Guo H., Yu H., Chen Y., Xu H., Zhao G. (2021). The role of the transcription factor EGR1 in cancer. *Frontiers in Oncology*.

[26] Zhang Y., Wang M., Zhang X. (2022). Helicid improves lipopolysaccharide-induced apoptosis of C6 cells by regulating SH2D5 DNA methylation via the CytC/Caspase9/Caspase3 signaling pathway. *Contrast Media & Molecular Imaging*.

[27] Jiang Q., Shan K., Qun-Wang X. (2016). Long non-coding RNA-MIAT promotes neurovascular remodeling in the eye and brain. *Oncotarget*.

[28] Shen Y., Dong L. F., Zhou R. M. (2016). Role of long non-coding RNA MIAT in proliferation, apoptosis and migration of lens epithelial cells: a clinical and in vitro study. *Journal of Cellular and Molecular Medicine*.

[29] Sattari A., Siddiqui H., Moshiri F. (2016). Upregulation of long noncoding RNA MIAT in aggressive form of chronic lymphocytic leukemias. *Oncotarget*.

